# Enhanced pup retrieval behaviour in a mouse model of polycystic ovary syndrome

**DOI:** 10.1111/jne.13206

**Published:** 2022-11-23

**Authors:** Zin Khant Aung, Renee R. Masih, Elodie Desroziers, Rebecca E. Campbell, Rosemary S. E. Brown

**Affiliations:** ^1^ Centre for Neuroendocrinology University of Otago Dunedin New Zealand; ^2^ Department of Physiology, School of Biomedical Sciences University of Otago Dunedin New Zealand; ^3^ Department of Anatomy, School of Biomedical Sciences University of Otago Dunedin New Zealand; ^4^ Sorbonne Université ‐ Faculté de Sciences et Ingénierie, Neuroplasticité des Comportements de la Reproduction, Neurosciences Paris Seine, UM119 ‐ CNRS UMR 8246 ‐ INSERM UMRS 1130 Paris France

**Keywords:** oestrogen receptor α, maternal behaviour, polycystic ovary syndrome, prenatal androgen, progesterone receptor

## Abstract

Polycystic ovary syndrome (PCOS) is the most common endocrinopathy to affect women of reproductive‐age world‐wide. Hyperandrogenism is both a hallmark feature of PCOS, and is hypothesised to be an underlying mechanism driving the development of the condition in utero. With circulating hormones known to profoundly influence maternal responses in females, we aimed to determine whether maternal behaviour is altered in a well‐described prenatally androgenised (PNA) mouse model of PCOS. Mouse dams were administered with dihydrotestosterone or vehicle on days 16, 17 and 18 of pregnancy. Maternal responses were assessed in both the dihydrotestosterone‐injected dams following parturition and in their adult female PNA offspring. Exposure of dams to excess androgens during late pregnancy had no detrimental effects on pregnancy outcomes, including gestation length, pup survival and gestational weight gain, or on subsequent maternal behaviour following parturition. By contrast, PNA virgin females, modelling PCOS, exhibited enhanced maternal behaviour when tested in an anxiogenic novel cage environment, with females rapidly retrieving pups and nesting with them. In comparison, most control virgin females failed to complete this retrieval task in the anxiogenic environment. Assessment of progesterone receptor and oestrogen receptor α immunoreactivity in the brains of virgin PNA and control females revealed increased numbers of oestrogen receptor α positive cells in the brains of PNA females in regions well known to be important for maternal behaviour. This suggests that increased oestrogenic signalling in the neural circuit that underlies maternal behaviour may be a possible mechanism by which maternal behaviour is enhanced in PNA female mice.

## Graphical abstract

Virgin female mice show enhanced pup retrieval behaviour in a mouse model of polycystic ovary syndrome. When tested in an anxiogenic novel cage environment, prenatally androgenised (PNA) female mice rapidly retrieve and nest with pups, with most control females failing to complete the retireval task. Assessment of progesterone receptor and oestrogen receptor alpha immunoreactivitiy in the brain of PNA and control mice, revealed increased number of oestrogen receptor alpha expressing cells in the brain of PNA gemales in regions well known to be important for maternal behaviour.

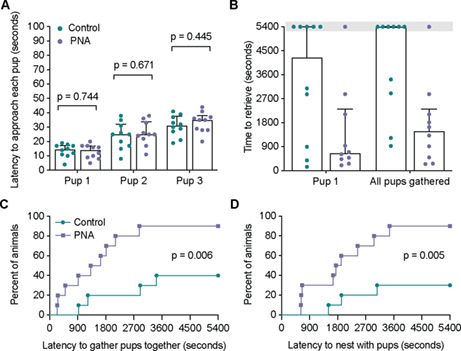

## 1 INTRODUCTION

Polycystic ovary syndrome (PCOS) is the most common endocrine disorder among women of reproductive age and affects approximately 6%–16% of women worldwide.^1^, ^2^ This condition, characterised by oligo‐ or anovulation, polycystic ovarian morphology and hyperandrogenism,^3^ is associated with high rates of sub or infertility as well as metabolic complications.^4^ Clearly, PCOS pathology involves several different organ systems, including the ovaries and brain.^5^, ^6^ A hallmark feature of PCOS,^7^ hyperandrogenism, is also hypothesised to be an underlying mechanism driving the development of the condition in utero.^8^, ^9^, ^10^ In women with PCOS, hyperandrogenaemia is found in 60%–80% of women, and hyperandrogenaemia remains significantly elevated during pregnancy.^11^, ^12^ Furthermore, the daughters of women with PCOS are more likely to be diagnosed with PCOS themselves,^13^ supporting the hypothesis that high androgen exposure during prenatal development contributes to PCOS development.^14^ The generation of animal models that successfully replicate the pathophysiology of PCOS has enabled investigation into the aetiology of this disease, and is most commonly modelled in animals through prenatal exposure to androgens during critical periods of development.^8^

In non‐human primates, sheep, rats and mice, prenatal androgen (PNA) exposure replicates many of the reproductive features of PCOS.^15^, ^16^, ^17^, ^18^ Within the animal models of PCOS, mice have become well‐utilised, with the generation of transgenic tools enabling detailed mechanistic studies to be undertaken. We and others have previously shown that dihydrotestosterone (DHT) treatment to late pregnant mice leads to development of the hallmark features of PCOS in the female offspring (PNA mice), including hyperandrogenism, disrupted oestrous cyclicity, markedly impaired fertility, altered ovarian morphology, impaired steroid hormone feedback and hyperactive gonadotropin secretion.^18^, ^19^, ^20^ PNA exposure in this model has been shown to significantly alter the neuronal network controlling reproduction.^20^ PNA is known to drive changes in steroid hormone receptor expression and changes in the organisation of GABAergic synaptic innervation and neurotransmission^20^ Altogether, this illustrates the ability of PNA exposure to induce significant changes to specific neural circuits.

In addition to reproductive dysfunction, it has more recently emerged that PCOS in women is also linked to a higher incidence of mood disorders including anxiety and depression.^4^, ^21^, ^22^ In mothers, PCOS is associated with higher incidence of postpartum depressed mood or anhedonia.^23^, ^24^ Increased androgen signalling in the brain is thought to underpin these features.^25^ In animal models of PCOS, behavioural disruptions have been comparatively understudied compared to reproductive and metabolic deficits. In both female mice and rats, prenatal DHT or testosterone treatment has been shown to result in increased anxiety‐like behaviour when tested in an elevated plus maze or open field.^26^, ^27^ Together, these human and animal studies warrant further investigation into the potential behaviour impacts of prenatal androgen excess associated with PCOS development. The focus of the present study is to investigate whether maternal behaviour, which is highly affected by levels of circulating hormones, is altered in an established mouse DHT‐treatment model of PCOS. Initially, we aimed to investigate whether deficits in maternal behaviour arise following DHT treatment in late pregnant mice. Second, we aimed to investigate whether maternal behaviour is disrupted in the female PNA offspring, which display PCOS‐like endocrine disruptions.

## 2 MATERIALS AND METHODS

### 2.1 Animals and tissue collection

Adult C57Bl/6 inbred female mice (age 8–16 weeks), originally sourced from Jackson Laboratory, were group housed under conditions of controlled temperature (22°C ± 1°C) and lighting (12:12 h light/dark photocycle, lights on 6:00 am), with ad libitum access to food and water. PNA mice for modelling PCOS were generated as previously described.^18^, ^19^ Briefly, two groups of pregnant dams were generated by mating individually‐housed 8–10‐week‐old wildtype female C57Bl/6 with C57Bl/6 stud males. The presence of a vaginal mucus plug was counted as day 1 of pregnancy, and the male was removed from the cage and the female individually housed throughout pregnancy and lactation. Females were weighed daily until gestation day 18. Pregnant dams received daily injections of either vehicle (sesame oil) or 250 μg of 5α‐androstan‐17β‐ol‐3‐one (free dihydrotestosterone, DHT; A8380; Sigma, St Louis, MO, USA) in sesame oil (s.c., 2.5 mg mL^−1^) on gestation days 16, 17 and 18 (*n* = 8 per group). Pregnant dams were weighed daily from day 1–18 of pregnancy, and day of parturition (counted as day 1 of lactation), as well as litter size at birth and at day 3 lactation, were recorded. Litter sizes were standardised to 5–7 pups on day 3 of lactation. Postpartum maternal behaviour of vehicle and DHT‐treated dams was assessed on days 3 and 5 of lactation.

**FIGURE 1:**
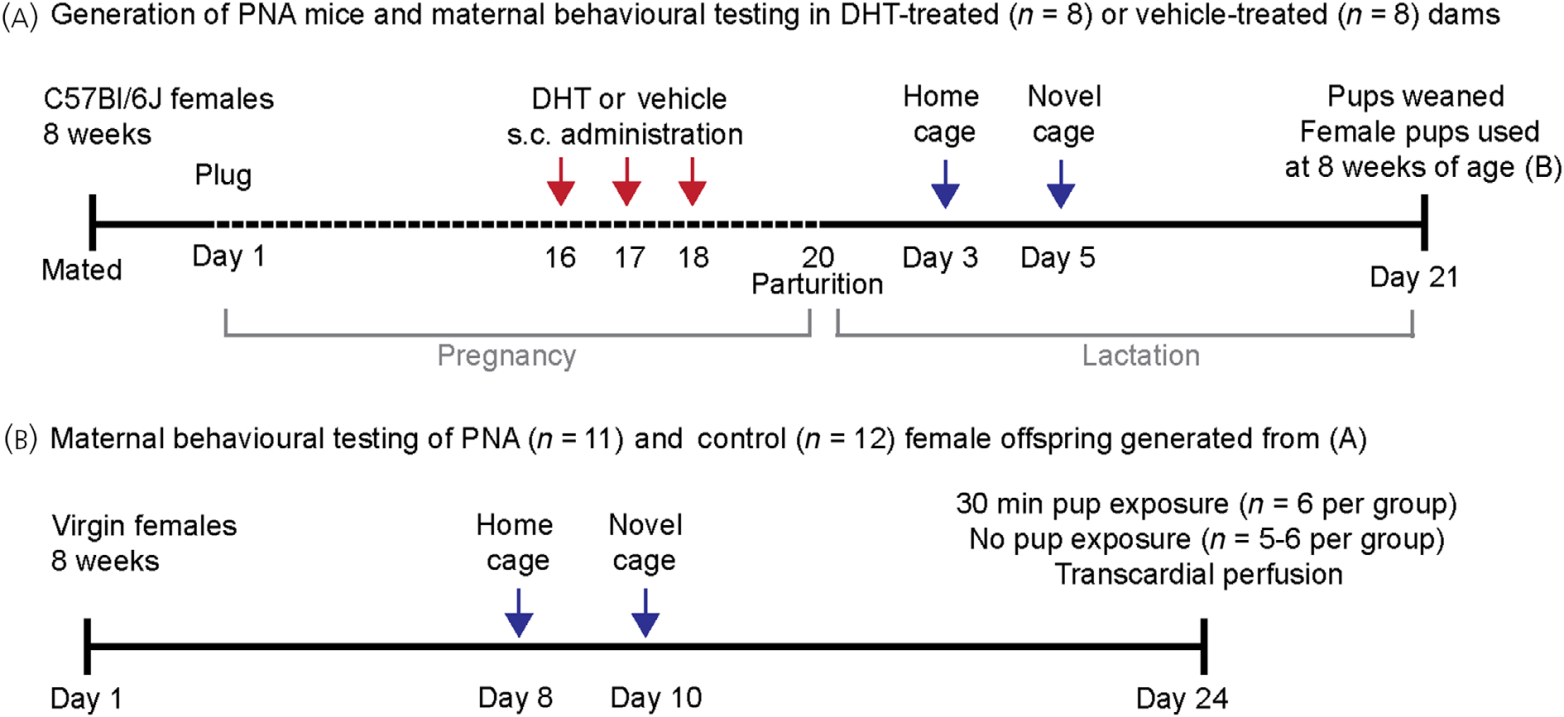
Experimental timeline of dihydrotestosterone (DHT) or vehicle treatment to pregnant dams and maternal behavioural testing. DHT or vehicle was administered (s.c.) on days 16, 17 and 18 of pregnancy (A). Day of parturition was counted as day 1 of lactation and maternal behaviour was tested in the home cage (day 3 of lactation) and novel cage (day 5 of lactation) (A). Pups from DHT or vehicle‐administered dams were weaned on day 21 of lactation and female offspring group housed until aged 8 weeks. At 8 weeks of age, females were individually housed and maternal behaviour tested in the home cage (day 8) and novel cage (day 10) (B). Two weeks following maternal behavioural testing, females were exposed to either foster pups or no pups and transcardially perfused 60 min following cessation of exposure (B)

Control and PNA offspring born to vehicle‐treated and DHT‐treated dams, respectively, were weaned on postnatal day 21 and the female offspring were group‐housed with littermates. At 8 weeks of age, control and PNA females (*n* = 11–12 per group) were individually housed, and oestrous cycles were monitored by daily vaginal cytology to confirm the acyclic phenotype of PNA mice. Vaginal cells were collected for cytological assessment between 9:00 and 10:00 am through aspiration of saline into the vagina. Cells were stained with Toluidine blue and cell morphology and density used to determine oestrous cycle state.^28^ At 9 weeks of age, maternal behaviour was assessed in virgin control and PNA offspring, and behaviour scored in 10 control and 10 PNA offspring (Figure 1). Two weeks following behavioural testing, six control and six PNA females were exposed to foster pups in the home cage for 30 min, and six control and five PNA female mice underwent the same procedure but no pups were introduced. Ninety min following introduction of foster pups, mice were anaesthetised with pentobarbital and transcardially perfused with 4% paraformaldehyde. Brains were removed, postfixed for 1 h in the same fixative and cryoprotected in 30% sucrose overnight. Brains were stored at −80°C until sectioned. The University of Otago Animal Ethics Committee approved all of the experimental procedures conducted with animals.

### 2.2 Maternal behaviour testing

Pup‐directed maternal responses were tested in both the home cage and in a novel cage. A novel cage is an anxiogenic environment and therefore, provides a more rigorous test of maternal behaviour.^29^, ^30^ We have previously reported that dams take longer to retrieve pups in a novel cage.^31^ All behavioural tests were recorded by video camera and subsequently scored by an experimenter who was blinded to treatment group.

### 2.3 Home cage pup retrieval test

Pup retrieval behaviour was tested in home cages on day 3 of lactation in vehicle and DHT‐treated dams and at 9 weeks of age in virgin control and PNA female offspring (Figure 1). Between 9:00 and 11:00 am, female mice were habituated to a testing room for 30 min and pups were removed from the home cages of lactating dams during testing. Three foster pups (3–5 days old; aged‐matched for lactating dams) were placed into the three corners of the cage at the opposite end from the nest and all pup‐directed behaviours were recorded for 30 min. Behaviours analysed during this test included latency to approach each pup (defined as the first time the nose of the female visually touched the pup), latency to retrieve each pup to the nest, grouping of pups in the nest and the time to reach kyphosis (arching of the back into a nursing position). In both a home and novel cage, virgin females did not assume kyphotic positions, as defined previously,^32^ but continued to move nesting material and investigate pups once in the nest, therefore latency to nest with pups has been reported rather than kyphosis. After testing, pups were returned to their home cages.

### 2.4 Novel cage pup retrieval test

Pup retrieval behaviour was tested in novel cages between 9:00 and 11:00 am on day 5 of lactation in dams and 2 days following home cage testing in virgin females (Figure 1). Females were placed in a clean, novel cage and allowed to habituate for 10 min. Three pups were placed into three corners of the cage and pup‐directed behaviours were recorded for 60 min in the dams and for 90 min in the virgin female groups. We have previously shown that virgin females display lower levels of maternal motivation to interact with pups^33^; therefore, they were given longer to complete the retrieval task. Behaviours analysed during this test included latency to approach and retrieve each pup, time taken to gather all three pups together and the time to reach kyphosis (only assessed for groups of lactating dams). In the absence of an established nest in the novel cage test, female mice frequently retrieved one pup to the location of another pup, without actively retrieving all pups. Therefore, for novel cage testing, only retrieval for the first pup, along with the latency to have all three pups gathered together, and latency to nest with pups has been reported. After testing, female mice were returned to their home cage.

### 2.5 Immunohistochemistry

To identify a potential mechanism by which PNA females show enhanced maternal behaviour, we investigated whether there were differences in pup exposure‐induced neuronal activation (measured by cFos immunolabelling) between PNA and control females. Immunohistochemical labelling for cFos has extensively been used as a marker of maternal behaviour‐induced neuronal activation.^34^, ^35^, ^36^, ^37^ Levels of cFos protein peak between 1 and 3 h following exposure to a stimuli^38^; therefore animals were transcardially perfused 90 min following first exposure to foster pups to evaluate cFos immunoreactivity. Previous studies have shown altered expression of progestrone (PR) and oestrogen receptor (ER)α in the brain of PNA female mice in areas associated with fertility regulation.^20^ With both progesterone and oestrogen known to be regulators of maternal behaviour, we also assessed whether there were changes in the immunolabelling of PR and ERα in regions of the brain known to regulate maternal behaviour.^39^, ^40^

Three sets of 30‐μm thick coronal sections through the forebrain from each animal were cut using a sliding microtome. Separate sets of brain tissue were used to label cFos (a marker of neuronal activation), PR and ERα by immunohistochemistry. To label cFos immunoreactivity, tissue was incubated in rabbit anti‐cFos primary antibody (dilution 1:5000; #190289; Abcam, Cambridge UK^37^) for 48 h at 4°C. Sections were then incubated in biotinylated goat anti‐rabbit IgG (dilution 1:200; Vector Laboratories, Burlingame, CA, USA) for 60 min, followed by a 90‐min incubation in Vector Elite avidin‐biotin‐horseradish peroxidase complex (dilution 1:100). Peroxidase labelling was visualised with nickel‐diaminobenzidine tetrahydrochloride using glucose oxidase to create a black nuclear precipitate. This procedure was replicated on separate sets of tissues to immunolabel PR and ERα, using a rabbit anti‐progesterone receptor primary antibody (dilution 1:2000; Dako Corp., Glostrup, Denmark^20^) and a rabbit anti‐ERα primary antibody (dilution 1:10,000; Millipore 06‐935; Merck, Darmstadt, Germany^20^), respectively. For each immunohistochemical experiment, negative controls were included where omission of primary antisera resulted in complete absence of the respective immunoreactivity.

Sections were analysed using a BX51 microscope (Olympus, Tokyo, Japan) equipped with a Gryphax NOS camera (Jenoptik, Jena, Germany) using brightfield microscopy using a 20× objective. Image settings were determined for each immunolabel and kept constant between sections and animals. Nuclei with positive immunolabelling were counted using ImageJ (NIH, Bethseda, MD, USA) bilaterally in each section and the experimenter was blinded to treatment group for analysis. Briefly, images were thresholded with threshold parameters determined for each immunolabel and kept constant between sections and animals. Positive immunolabelling was identified using the analyse particle function in two anatomically‐matched sections per region per animal, with the average nuclei counts in the region of interest reported per 30‐μm thick section.^20^, ^37^, ^41^ Analysis of cFos immunolabelling was undertaken in the anteroventral periventricular area (AVPV) (Bregma 0.26–0.38 mm), medial preoptic area (MPOA) (Bregma −0.10 to 0.14 mm), bed nucleus of the stria terminals (BNST) (Bregma −0.10 to −0.22 mm) and the medial amygdala (MEA) (Bregma −1.94 to −2.06 mm).^42^ Within the BNST, cFos and ERα immunolabelling was observed in the medial division, ventral part of the medial division, posteromedial part of the medial division, dorsal part of the lateral division and the ventral part of the lateral division. For analysis of positive cFos and ERα immunoreactivity, labelling from all these subdivisions was counted in the BNST. Within the medial amygdala, immunolabelling for cFos and ERα were observed and counted in both the posteroventral and posterodorsal medial amygdala. These regions were selected based on assessment of immunolabelling present and previous reports of pup interaction‐induced cFos immunolabelling in the rodent brain.^36^ Analysis of PR immunolabelling was undertaken in the AVPV, MPOA, arcuate nucleus (Bregma −1.70 to −1.94 mm) and ventromedial hypothalamic nucleus (Bregma −1.82 to −1.94 mm). Very few nuclei that were immunopositive for PR were observed in the BNST or MEA; therefore, cell counts are not reported in this regions. Analysis of ERα immunolabelling was undertaken in the AVPV, MPOA, BNST and MEA.

### 2.6 Statistical analysis

Statistical analysis was performed using Prism, version 9 (GraphPad Software Inc., San Diego, CA, USA). Data are presented as the mean ± SEM for normally distributed data and the median ± interquartile range for non‐normally distributed data. *p* < .05 was considered statistically significant. All data sets (apart from latency measures) were first tested for normal distribution using the Shapiro–Wilk normality test, where *p* < .05 was considered as not normally distributed. Normal distribution was confirmed for weight gain during pregnancy, pup‐directed approach behaviour of virgins in the novel cage, and PR and ERα immunolabelling. When a normal distribution was confirmed, further analysis was performed using parametric unpaired two‐tailed *t* tests with Welch's correction (to account for differing population variance or sample size). Normal distribution was not confirmed for gestation length, number of pups, percent of time in oestrous cycle stage, pup‐directed approach and retrieval behaviour, and gathering of pups by dams in home and novel cages and for virgins in the home cage. Therefore, all of these data were analysed using the non‐parametric Mann–Whitney test. Latency to gather pups to nest and to reach kyphosis were analysed using survival analysis as a result of the high proportion of censored data (animals that failed to complete the task within the duration of the test), and curve comparison was undertaken using the Mantel–Cox log‐rank test. The proportion of control and PNA females successfully gathering pups together in the novel cage was analysed by Fisher's exact test. Pup‐exposure induced differences in cFos immunoreactivity between control and PNA females were compared in each brain region by two‐way analysis of variance (ANOVA) and Tukey's multiple comparison test was used to identify statistically significant changes. N min of pup exposure had no effect on levels of PR or ERα immunoreactivity; therefore, pup‐exposed (*n* = 6 per group) and non‐exposed (*n* = 5–6 per group) groups were combined to compare levels of immunoreactivity between control and PNA females using parametric unpaired two‐tailed *t* tests with Welch's correction. For each brain region, the comparison of interest was the immunoreactive cell counts between PNA and control groups. Different brain regions were not compared with each other. Full statistical tables are provided in the Supporting information (Tables S1).

## 3 RESULTS

### 3.1 DHT‐treatment during late pregnancy does not affect pregnancy outcomes

**FIGURE 2:**
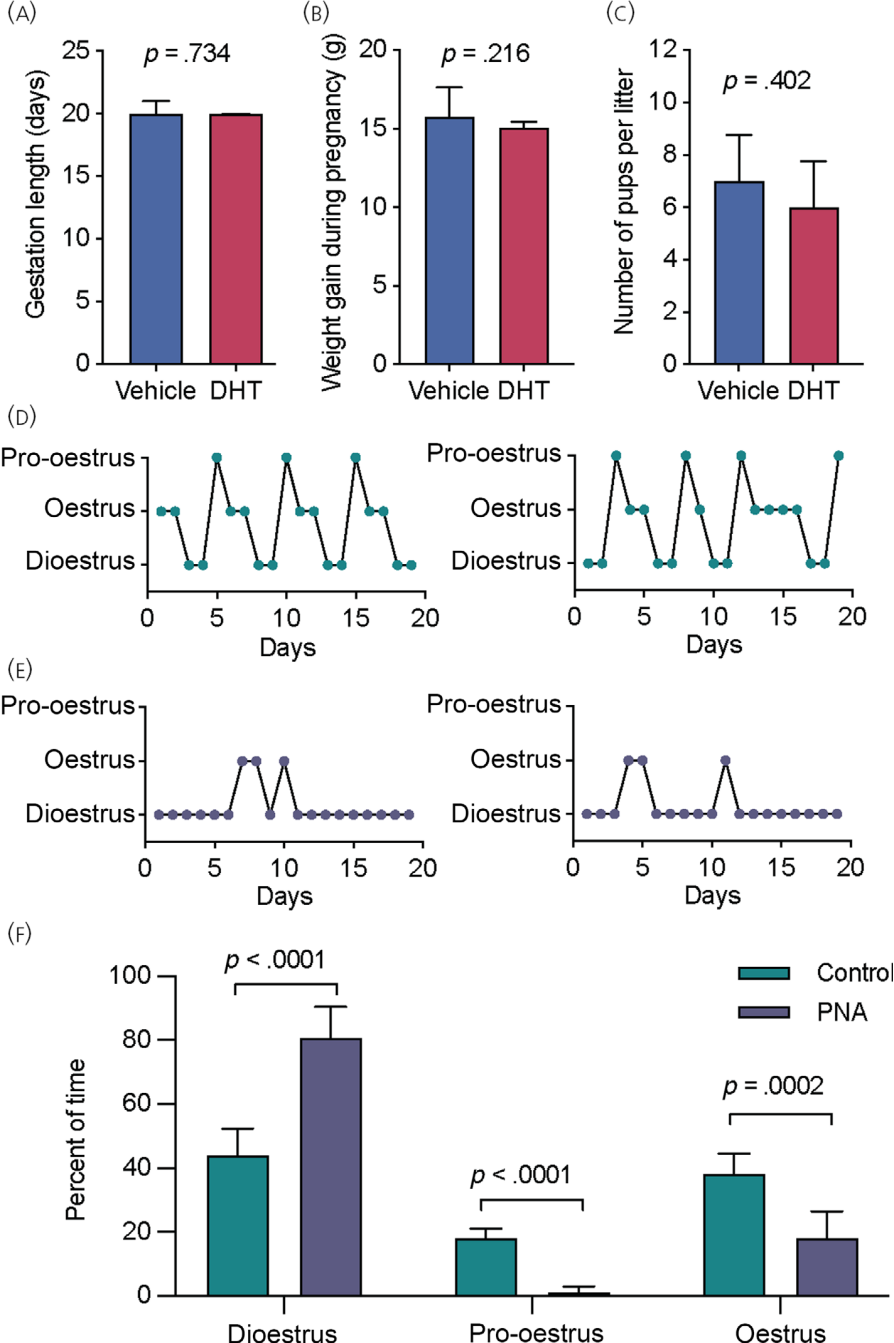
Dihydrotestosterone (DHT) treatment during late pregnancy has no detrimental effects on pregnancy outcomes in a mouse model of polycystic ovary syndrome (PCOS). DHT treatment on days 16, 17 and 18 of pregnancy had no effect on gestation length (A; median ± interquartile range [IR]), total weight gain during pregnancy (B; median ± IR) or number of live pups per litter on day three of lactation (C; median ± confidence interval [CI]) (*n* = 8 per group). DHT treatment led to the successful generation of PNA female offspring (D–F; mean ± 95% CI). Adult control females (green) showed normal oestrous cycles, with severely disrupted cycles observed in prenatally androgenised (PNA) females (purple) (*n* = 10 per group)

To assess whether DHT‐treatment during pregnancy days 16–18 detrimentally affected pregnancy outcomes, gestation length, pregnancy weight gain and pup survival were monitored. There were no differences in any of these parameters between vehicle‐treated and DHT‐treated pregnant females (Figure 2A–C; see also Supporting information, Table S1). Successful DHT treatment to model PCOS was confirmed by the marked disruption in oestrous cyclicity that was observed in female PNA offspring once they reached 8–10 weeks of age compared to control females (offspring of vehicle‐treated pregnant dams) (Figure 2D–F; see also Supporting information, Table S1). Consistent with previous reports,^18^, ^19^ PNA females spent significantly m time in diestrus compared to control females (80.70% ± 4.21% vs. 44.01% ± 1.92%, respectively; *p* < .0001, Mann–Whitney test) and pro‐oestrous cytology was rarely observed in only two out of 10 PNA females (Figure 2F).

### 3.2 DHT‐treatment during late pregnancy does not adversely affect maternal behaviour during lactation

**FIGURE 3:**
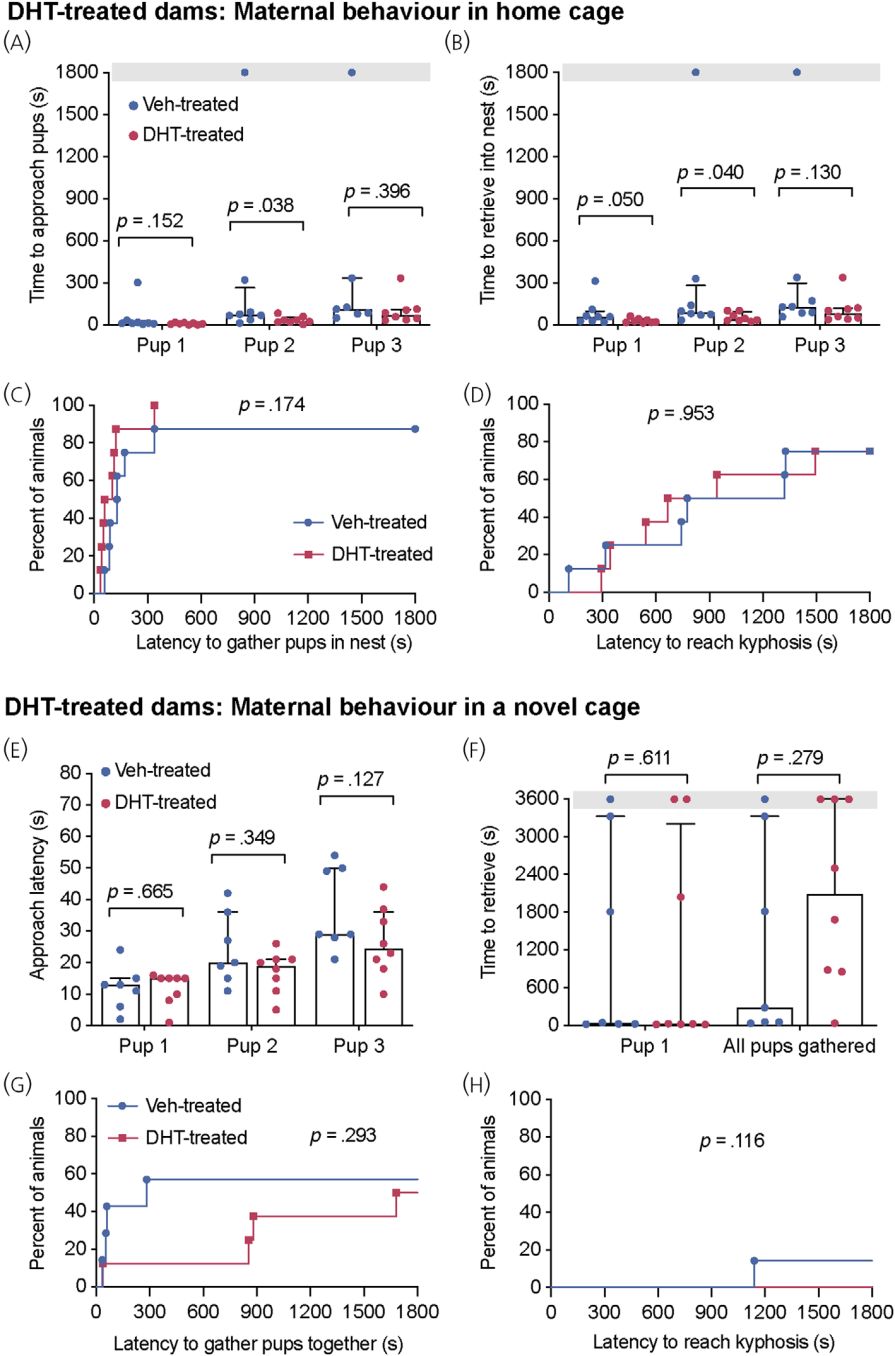
No disruption of maternal behaviour following dihydrotestosterone (DHT)‐treatment to pregnant dams. Maternal behaviour was tested in the home cage on day 3 of lactation (A–D) and in a novel clean cage on day 5 of lactation (E, F) in groups of mice treated with vehicle or DHT during late pregnancy (*n* = 8 per group). Mean latency to approach (A, E; median ± interquartile range [IR]) and retrieve (B, F) each pup was recorded and cumulative percentage of mice from each group to gather pups together (C, G) and reach kyphosis (D, H). Grey bars in (A), (B) and (F) indicate the length of each test where at least one animal failed to complete the behavioural task

Postpartum maternal behaviour was tested in dams that received either vehicle‐ or DHT‐treatment in late pregnancy. First, pup‐directed maternal responses were examined in the home cage on day 3 of lactation. All dams approached and successfully retrieved the first foster pup to the nest, with only one vehicle‐treated female failing to approach and retrieve the second and third foster pups (Figure 3A,B; see also Supporting information, Table S2). Although, the DHT‐treated dams were significantly faster to approach the second pup and to retrieve the first and second pups than the vehicle‐treated dams, this is likely a result of the failure of one dam (out of eight) to approach and retrieve pups in the vehicle‐treated group (Figure 3A,B). There were no significant differences in the latency to gather all three foster pups to the nest or to reach kyphosis (Figure 3C,D; see also Supporting information, Table S2). On day 5 of lactation, pup‐directed maternal responses were tested in a novel cage, with the anxiogenic environment being a more rigorous test of maternal behaviour.^29^ As reported previously,^31^ dams took longer to retrieve pups, with more dams failing to complete this task in a novel cage (Figure 3E–H). There were no differences between vehicle‐ and DHT‐treated dams in the time taken to approach and retrieve pups or to gather pups in the nest (Figure 3E–G; see also Supporting information, Table S2). In this novel environment, only two out of eight vehicle‐treated dams reached kyphosis in the duration of the 60 min test (Figure 3H; see also Supporting information, Table S2). Together, these data suggest that DHT‐treatment during pregnancy does not adversely affect postpartum maternal behaviour.

### 3.3 PNA virgin females display enhanced maternal responses in a novel environment

**FIGURE 4:**
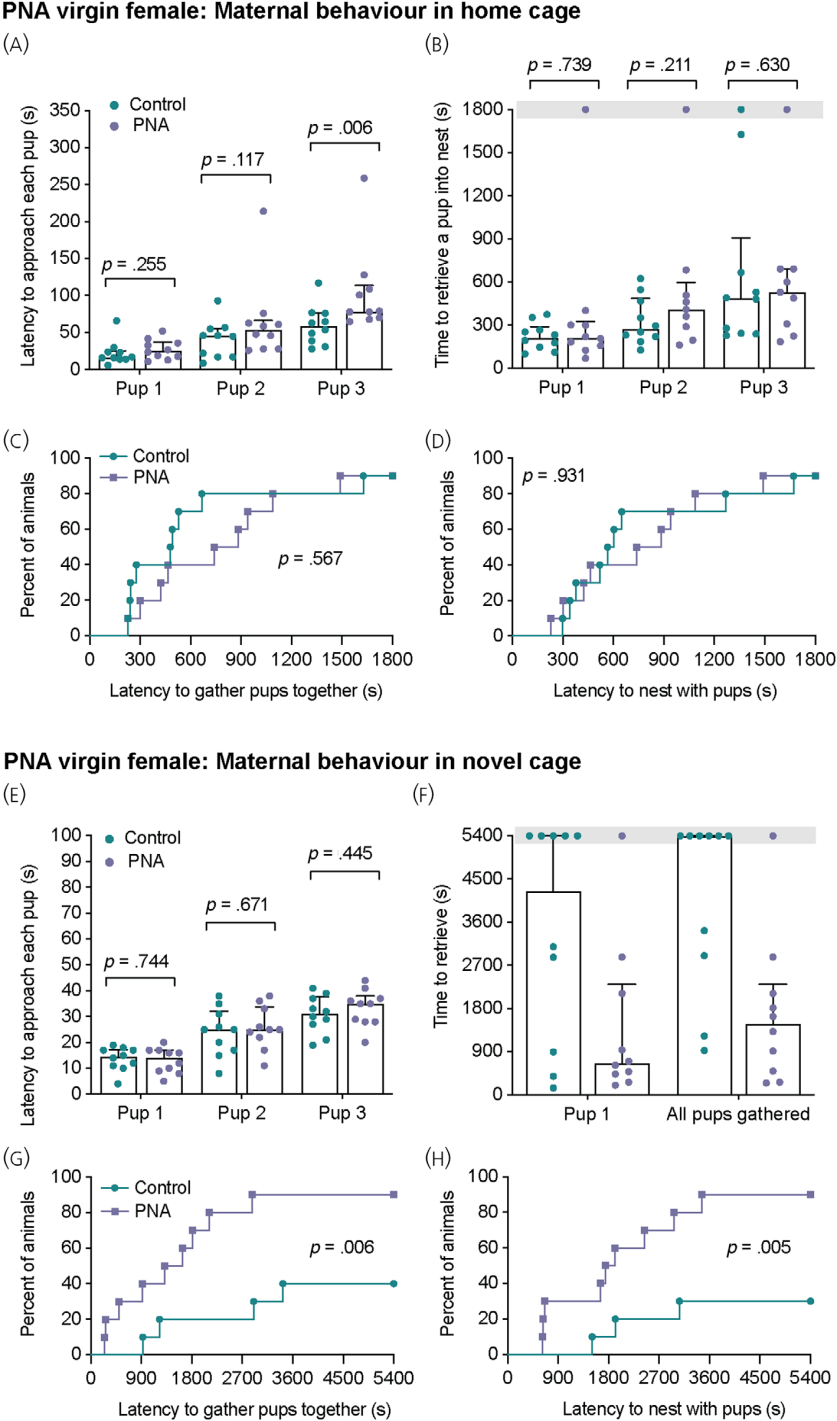
Enhanced pup retrieval behaviour by adult virgin PNA females in a novel cage. Maternal behaviour was tested in the home cage (A–D) and 2 days later, in a novel clean cage (E–H) in groups of control and PNA female mice (*n* = 10 per group). Mean latency to approach (A, E; median ± interquartile range [IR]) and retrieve (B, F; median ± IR) each pup was recorded and cumulative percentage of mice from each group to nest with the pups (D, H). Note that only five of 10 and four of 10 of control females successfully retrieved one pup and gathered the pups together in the novel cage, compared to nine of 10 PNA females (F, G). Grey bars in (B) and (F) indicate the length of each test where at least one animal failed to complete the behavioural task

The impact of prenatal androgen exposure on subsequent adult maternal behaviour was assessed in virgin PNA female mice in response to foster pups. Although it appears that reproductive function may be rescued following long‐term anti‐androgen treatment,^18^, ^43^ the vast majority of PNA mice are infertile. Therefore, we aimed to investigate whether the infertile, untreated, hyperandrogenic PCOS‐like model was accompanied by altered maternal behavioural responses to foster pups. When tested in the home cage, pup‐directed maternal behaviour was only subtly different between PNA and control females (Figure 4A–D; see also Supporting information, Table S3). PNA females were significantly slower to approach the third pup; however, there were no differences in the time taken to retrieve all three pups or to gather pups in the nest between groups (Figure 4A–D; see also Supporting information, Table S3). In both PNA and control groups, nine out of 10 females retrieved all three pups to the nest (Figure 3C). Surprisingly, when tested 2 days later in a novel cage, PNA females showed markedly higher levels of maternal behaviour than control females (Figure 4E–H; see also Supporting information, Table S3). Although pup approach was not different between groups, only five out of 10 control females retrieved the first pup compared to nine out of 10 PNA females (Figure 4F,G). PNA females showed reduced latencies to retrieve the first pup and gather all pups in the nest compared to control females (Retrieval of first pup: χ^2^ = 4.173, *df* = 1, *p* = .0411; all pups gathered: χ^2^ = 7.541, *df* = 1, *p* = 0.006; Mantel–Cox log‐rank tests). PNA females also showed much reduced latencies to nest with the foster pups (χ^2^ = 8.060, *df* = 1, *p* = .005; Mantel–Cox log‐rank tests), with significantly more PNA females completing this behaviour within the duration of the test compared to control females (nine out of 10 PNA females compared to three out of 10 control females, *p* = .0198, Fisher's exact test) (Figure 4H).

### 3.4 Pup exposure induces cFos immunoreactivity in both PNA and control females, with no difference between these groups

**FIGURE 5:**
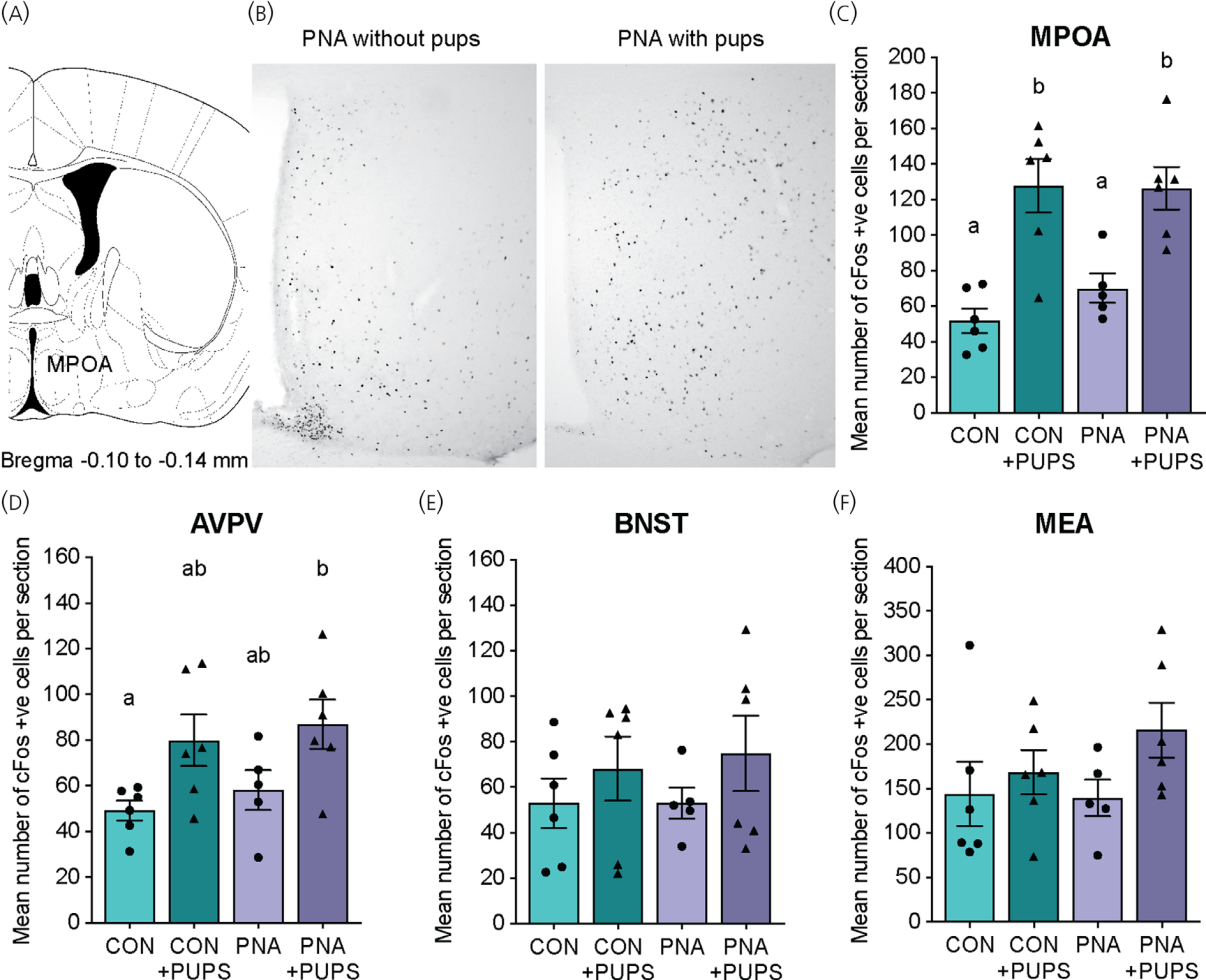
Pup‐induced cFos immunoreactivity in prenatally androgenised (PNA) and control female mice. (A, B) Representative sections showing increased levels of cFos immunoreactivity in the medial preoptic area (MPOA) of PNA females following 30 min of pup exposure. Mean number of cFos‐positively labelled cells following pup exposure or no exposure in groups of control and PNA females (*n* = 5–6 per group) in the MPOA (C), anteroventral periventricular area (AVPV) (D), bed nucleus of the stria terminalis (BNST) (E) and medial amygdala (MEA) (F). Data are shown as the mean ± SEM. In the graphs, groups with different letters are significantly different (*p* < .05), as determined by two‐way analysis of variance and Tukey's multiple comparison tests

In both control and PNA females, pup exposure in the home cage induced a significant increase in cFos immunoreactivity in the MPOA, from 51.8 ± 6.8 to 127.7 ± 15.1 positively labelled cFos nuclei following pup exposure in control mice and from 70.1 ± 8.2 to 126.4 ± 12.1 positively labelled cFos nuclei in PNA females (two‐way ANOVA, no effect of DHT treatment *p* = .462; significant effect of pup exposure, *p* < .0001; no significant interaction, *p* = .398) (Figure 5A–C; see also Supporting information, Table S4). No significant differences in cFos immunolabelling following pup exposure were observed in the AVPV, BNST or MEA in either control of PNA mice (Figure 5D–F; see also Supporting information, Table S4). There were no differences in basal levels (in the absence of pup exposure) of cFos immunolabelling between control and PNA females in any of the regions examined.

### 3.5 PNA virgin females show higher levels of oestrogen receptor α but not progesterone receptor immunoreactivity in brain regions known to be important in mediating aspects of maternal behaviour

**FIGURE 6:**
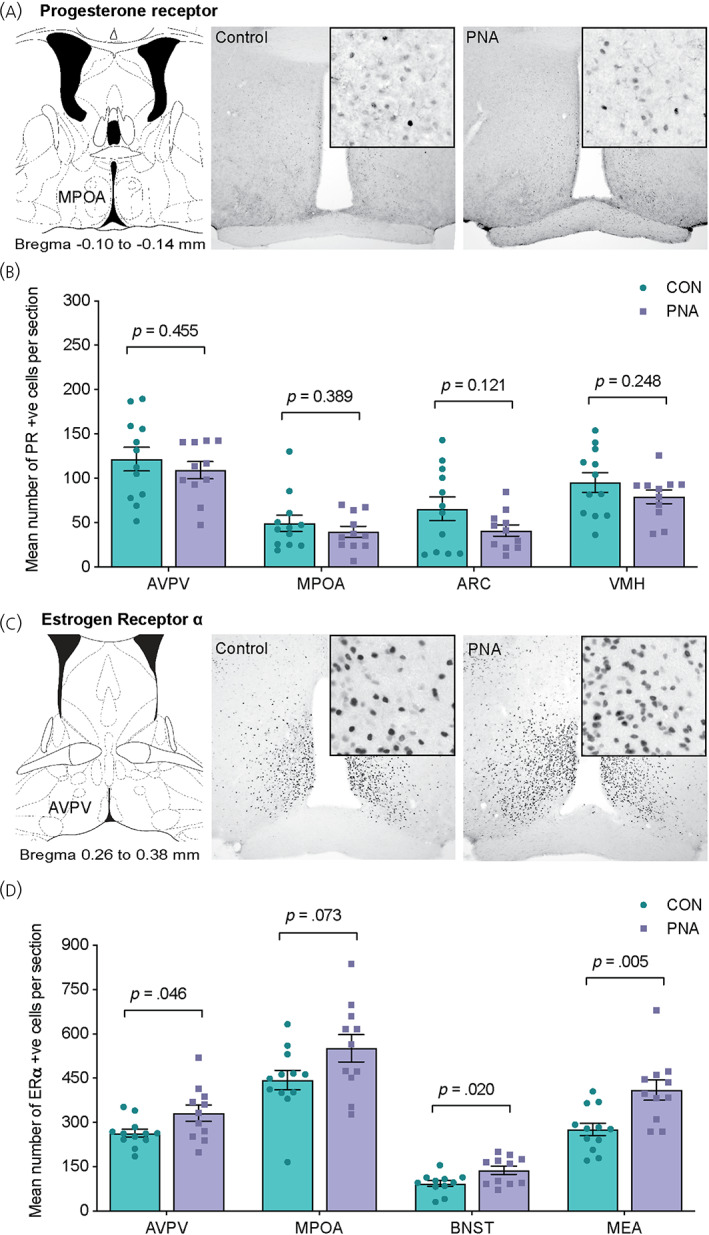
Progesterone receptor and oestrogen receptor α immunoreactivity in prenatally androgenised (PNA) and control female mice. (A) Representative sections showing progesterone receptor (PR) immunoreactivity in the medial preoptic area (MPOA) of a control and a PNA virgin female mouse. (B) Mean number of PR‐positively labelled cells in groups of control and PNA females (*n* = 11–12 per group) in the anteroventral periventricular area (AVPV), MPOA, arcuate nucleus (ARN) and ventromedial nucleus of the hypothalamus (VMN). (C) Representative sections showing oestrogen receptor α (ERα) immunoreactivity in the AVPV of a control and a PNA virgin female mouse. (D) Mean number of ERα‐positively labelled cells in groups of control and PNA females (*n* = 11–12 per group) in the AVPV, MPOA, bed nucleus of the stria terminalis (BNST) and medial amygdala (MEA). Data are shown as the mean ± SEM

With progesterone and oestrogen action on the maternal neural circuit known to be important for regulating maternal behaviour, we subsequently investigated whether there were differences in PR and ERα immunolabelling between control and PNA females. PR immunolabelling was observed in the AVPV, MPOA, ARC and VMH (no labelling was observed in the BNST or MEA), but there were no significant differences between control and PNA female mice (Figure 6A,B; see also Supporting information, Table S5). By contrast, significant differences in ERα immunolabelling were detected in the AVPV, BNST and MEA, with higher levels found in PNA compared to control females (Figure 6C,D; see also Supporting information, Table S5).

## 4 DISCUSSION

The present study aimed to investigate whether changes in maternal behaviour occur in the PNA mouse model of PCOS. We initially investigated the pregnant dams in which the PNA model is produced and found that exposure to the non‐aromatisable androgen DHT during late pregnancy has no detrimental effects on their pregnancy outcomes or on their subsequent maternal behaviour following parturition. Importantly, this implies that any altered behaviours in the offspring are unlikely to result from altered maternal care that they received as pups, but rather to be a consequence of their own PNA programming and phenotype. Unexpectedly, in the PNA offspring that model PCOS, we observed enhanced maternal behaviour. In an anxiogenic environment, PNA virgin females rapidly retrieved pups and spent increased time with them in the nest. Our data showed that, although the general activation of maternal behaviour‐related brain circuits was not different in the brains of virgin PNA female mice, ERα immunoreactivity was increased, suggesting a possible mechanism by which maternal behaviour is altered in PNA female mice. It is important to note that maternal behaviour in the female PNA offspring was only evaluated in virgin females (as a result of impaired fertility in this model). It is not currently known whether postpartum maternal behaviour in the PNA female offspring would be altered in this model.

The enhanced maternal behaviour observed in PNA female mice was only observed when tested in a novel and clean cage. Unlike rats, virgin female mice of most strains have been shown to rapidly retrieve pups in a home cage,^44^ and it is unlikely that further improvements in this behaviour would be detectable under these conditions. For example, we have previously reported impaired retrieval behaviour in dams with a GABA neuron‐specific deletion of the prolactin receptor, although this was only observed in a novel but not the home cage.^31^, ^33^ In both rat and mouse PCOS models that utilise DHT‐treatment in late pregnancy, the female PNA offspring have been shown to display increased anxiety‐like behaviour in an elevated plus maze.^26^, ^27^, ^45^ This anxiety‐like phenotype of PNA females in a mouse model of PCOS is consistent with human data showing increased rates of anxiety in women with PCOS.^21^, ^46^ Although not assessed in the present study, given that the mouse strain, DHT‐treatment regime and behavioural testing conditions were consistent with these prior mouse studies,^26^, ^45^ we would predict that anxiety‐related behaviour would also have been elevated in the PNA female mice used in the present study. The observed increased maternal behaviour in an anxiogenic environment in the present study is therefore more remarkable, and indicates that maternal behaviour is specifically increased in this PCOS model rather than resulting from an indirect effect mediated through lowered anxiety. It would be of interest for future studies to examine whether increased maternal motivation, the high responsiveness to offspring‐related stimuli combined with a strong drive to seek out these stimuli,^47^, ^48^, ^49^ is underlying the enhanced maternal behaviour of PNA female mice.

With oestrogens known to be an important regulator of maternal behaviour,^39^, ^40^ one potential mechanism that could explain enhanced maternal behaviour in PNA female mice is increased levels of ERα expression. In the present study, we showed increased numbers of ERα positive immunoreactive cells in the AVPV, BNST and MEA of PNA female mice, with all these regions known to be important in regulating maternal behaviour. The role of oestrogen signalling in maternal behaviour is most well characterised in the MPOA, where we saw a trend but not a significant increase in ERα immunoreactivity in PNA females. In the MPOA, ERα positive cells project to the ventral tegmental area (VTA), part of the reward circuitry of the brain, and are important mediators of pup approach and retrieval behaviours.^50^ Although not investigated in the present study, little or no ERα expression has been reported in the VTA of female rodents,^51^, ^52^ suggesting that oestrogen action through ERα in the VTA would need to occur via an indirect mechanism. In the BNST and MPOA, a clear correlation has been shown between low numbers of ERα positive cells and poor maternal behaviour in a mandarin vole model of early life deprivation.^53^

In addition to retrieval behaviour, oestrogen signalling also appears to be important for nest building behaviour, with oestradiol implants in the MPOA and BNST stimulating maternal nest building behaviour in rabbits.^54^ In light of these studies, increased ERα signalling is one potential mechanism underlying the enhanced retrieval and nesting behaviour of PNA females seen in the present study. It is important to note that, although ERα immunoreactivity was reported to increase collectively in the BNST in the present study, immunoreactivity was observed in multiple subdivisions of the BNST. Oestrogen action through differential ERα expression in specific subdivisions of the BNST in PNA mice could be altering selective aspects of maternal behaviour, with the subdivisions having distinct neural connections and physiological roles.^55^ It has also recently been reported that progesterone exposure alone is able to induce pregnancy‐like nest building behaviour through actions mediated by cocaine‐ and amphetamine‐regulated transcript neurons in the Edinger–Westphal nucleus.^56^ Although PR immunolabelling in the Edinger–Westphal nucleus was not studied in the present study, we observed no change in PR immunoreactivity in any of the other brain regions examined. This suggests that altered progesterone receptor expression is unlikely to be contributing to altered maternal behaviour in PNA mice in the present study. We also observed increased ERα immunoreactivity in the MeA of PNA virgin females. Although lesioning studies led to the long‐held belief that the MeA suppresses maternal behaviour,^57^, ^58^, ^59^ a recent study showed that optogenetic stimulation of a subset of MeA neurons can induce pup grooming and retrieval behaviour.^60^ It would be of interest to investigate whether there is a role for ERα signalling within the MeA on promoting pup‐directed responses. Increased oestrogen signalling could also be acting indirectly to promote maternal behaviour in PNA female mice. It has previously been shown that oestradiol administration enhances the anxiolytic actions of oxytocin,^61^ and increases levels of oxytocin receptor binding in the MPOA.^62^ Together, these studies suggest that further investigation into whether elevated levels of ERα immunoreactivity can facilitate improved maternal behaviour are warranted.

Accompanying the increase in ERα expression, there may also be an increase in the availability of oestradiol to act on the receptor. Replicating that seen in women with PCOS, PNA female mice have high levels of circulating testosterone,^18^, ^19^ which, in the presence of aromatase, is converted into oestradiol. Aromatase expression has been reported in the BNST, MPOA and MeA of both male and female adult mice, with the highest numbers of aromatase‐expressing cell bodies in females located in the BNST and MeA.^63^ Interestingly, in both the BNST and MEA, it was reported that 60% of aromatase‐expressing cells in females also co‐express ERα.^63^ In ovariectomised mice, treatment with the aromatase inhibitor, letrozole, impaired pup retrieval and nest building behaviour,^64^ suggesting that local oestrogen production is contributing to maternal behaviour. Investigations into aromatase activity throughout the brain of PNA mice could provide valuable insights into whether local increases in oestrogen signalling may be occurring. Altered ERα signalling could also account for why enhanced maternal behaviour was seen in PNA offspring, but not in the DHT‐treated dams. The treatment of dams in the present study, with the non‐aromatisable DHT, specifically increased androgen receptor‐mediated effects (or androgen actions), without necessarily changing circulating oestrogen levels or local oestrogen production through aromatisation.

In addition to progesterone and oestrogen, androgens also modulate maternal behaviour. With hyperandrogenism being a key feature in clinical PCOS and the basis for most preclinical animal models of the disorder, it is important to consider potential effects of elevated androgens on mammalian maternal behaviour. A recent RNA‐sequencing analysis of the MPOA of lactating rats with and without pups, showed that androgen receptor (AR) expression is downregulated in the presence of pups.^65^ In that same study, central administration of an AR antagonist to rat dams increased pup‐directed behaviour,^65^ suggesting that AR‐signalling in the brain suppresses normal postpartum maternal behaviour. To date, few studies have investigated the impact of androgen administration during pregnancy on subsequent maternal behaviour in mammals. One study, conducted in the 1970s, administered testosterone during mid‐late gestation in rabbits, and reported no impact on postpartum maternal behaviour.^66^ Consistent with this finding by Anderson et al.,^66^ impaired maternal behaviour in dams following late pregnancy administration of DHT was not observed in the present study, suggesting that postpartum circulating levels of androgens are unlikely to be elevated in these mice. In apparently contradictory findings, co‐treatment of testosterone and progesterone to virgin female rats has been shown to increase pup‐directed behaviour.^67^ However, this appeared to occur via an oestrogen‐dependent mechanism, rather than directly mediated by androgens, because increased pup‐directed behaviour was blocked in the presence of an aromatase inhibitor.^67^ In the female offspring of mothers exposed to elevated androgens during pregnancy, poor nest building behaviour has been reported in adult virgin rabbits.^66^ Nest building was not investigated in the present study, but pup‐directed behaviour was clearly not negatively affected in PNA female mice, despite the hyperandrogenism phenotype in this PCOS model.^19^, ^43^

Together, these data provide the first description of maternal behaviour both in mouse dams treated with DHT during pregnancy and in the female PNA offspring. Such data can provide confidence to researchers employing prenatal androgen excess models with respect to the maternal behaviour of exposed dams not being compromised. In addition, enhanced pup retrieval behaviour in virgin PNA females when tested in an anxiogenic environment was an interesting observation especially given the anxiety‐like behaviour reported in this model that mirrors the elevated frequency of anxiety in women with PCOS. Our findings in the brain suggest enhanced signalling through ERα in regions of the brain important for maternal behaviour may underlie these responses.

## AUTHOR CONTRIBUTIONS

**Zin Khant Aung:** Data curation; formal analysis; investigation. **Renee R Masih:** Data curation; formal analysis; investigation; methodology; writing – original draft. **Elodie Desroziers:** Conceptualization; funding acquisition; supervision; writing – review and editing. **Rebecca E Campbell:** Conceptualization; methodology; supervision; writing – review and editing. **Rosemary Shanon Eileen Brown:** Conceptualization; data curation; formal analysis; funding acquisition; methodology; project administration; resources; supervision; writing – original draft; writing – review and editing.

## CONFLICTS OF INTEREST

The authors declare that they have no conflicts of interest.

## Supporting information


**Table S1.** Pregnancy outcomes following DHT treatment to pregnant times and oestrous cycles in female PNA offspring. Statistically significant *p* values indicated in bold (*p* < .05).

**Table S2.** Maternal behaviour tested in the home cage and novel cage in dams following DHT or vehicle treatment during late pregnancy. Statistically significant *p* values indicated in bold (*p* < .05).

**Table S3.** Maternal behaviour in response to foster pups in adult virgin control and PNA females when tested in the home cage and novel cage. Statistically significant *p* values indicated in bold (*p* < .05).

**Table S4.** Pup‐induced cFos immunoreactivity in the brains of control and PNA females. Statistically significant *p* values indicated in bold (*p* < .05).

**Table S5.** Progesterone receptor and oestrogen receptor α immunoreactivity in the brains of control and PNA females. Statistically significant *p* values indicated in bold (*p* < .05).

## Data Availability

The data that support the findings of this study are available from the corresponding author upon reasonable request.
